# Photoresponsive Block Copolymers Containing Azobenzenes and Other Chromophores

**DOI:** 10.3390/molecules15010570

**Published:** 2010-01-26

**Authors:** Haifeng Yu, Takaomi Kobayashi

**Affiliations:** 1 Top Runner Incubation Center for Academia-Industry Fusion, Nagaoka University of Technology, 1603-1 Kamitomioka, Nagaoka 940-2188, Japan; 2 Department of Materials Science and Technology, Nagaoka University of Technology, 1603-1 Kamitomioka, Nagaoka 940-2188, Japan; E-Mail: takaomi@nagaokaut.ac.jp

**Keywords:** photoresponsive block copolymers, microphase separation, azobenzenes, liquid crystals, advanced materials

## Abstract

Photoresponsive block copolymers (PRBCs) containing azobenzenes and other chromophores can be easily prepared by controlled polymerization. Their photoresponsive behaviors are generally based on photoisomerization, photocrosslinking, photoalignment and photoinduced cooperative motions. When the photoactive block forms mesogenic phases upon microphase separation of PRBCs, supramolecular cooperative motion in liquid-crystalline PRBCs enables them to self-organize into hierarchical structures with photoresponsive features. This offers novel opportunities to photocontrol microphase-separated nanostructures of well-defined PRBCs and extends their diverse applications in holograms, nanotemplates, photodeformed devices and microporous films.

## 1. Introduction

In the past two decades, photoresponsive polymers (PRPs) containing azobenzenes (AZ) and other chromophores in the side or main chains have been extensively studied as advanced materials with photoresponsive characteristics, many different aspects of which have been reviewed in [1,2,3,4,5,6,7,8,9,10,11]. Most of their excellent properties are based on photoresponsive behaviors, including photoisomerization, photocrosslinking (or photodimerization), photoalignment, and photoinduced cooperative motions. Incorporating a PRP as one of constituent segments into block copolymers (BCs), the obtained photoresponsive BCs (PRBCs) is expected to bring the photoresponsive feature of PRPs into the microphase separation of well-defined BCs. Especially, if the PRP block shows a mesogenic phase, the regular periodicity of liquid-crystalline (LC) ordering will influence on the microphase-separated nanostructures, making it possible to self-assemble into periodic nanostructures on a macroscopic scale [12,13,14].

PRBCs are one of novel macromolecules of industrial and academic interest, because they offer opportunities for the study of the formation and control of organic nanostructures under the influence of more than one driving force. Generally, such microphase-separated nanostructures should affect functionality (e.g., photoresponse), and *vice versa*, and specific functionalities (e.g., photoresponse and LC properties) should exert influence on the diverse nanostructures of PRBCs [14]. In PRBCs showing LC properties, the interplay between the microphase separation and the elastic deformation of LC ordering can be defined as supramolecular cooperative motion (SMCM) [12]. It has been shown that the SMCM enables PRBCs to exhibit more hierarchical structures with photoresponsive features, which offers novel methods to control supramolecularly self-assembled nanostructures [12,13]. Combining photoresponsive properties of a PRP with microphase separation of BCs, PRBCs can promise to find their diverse applications in advanced technologies. 

**Figure 1:**
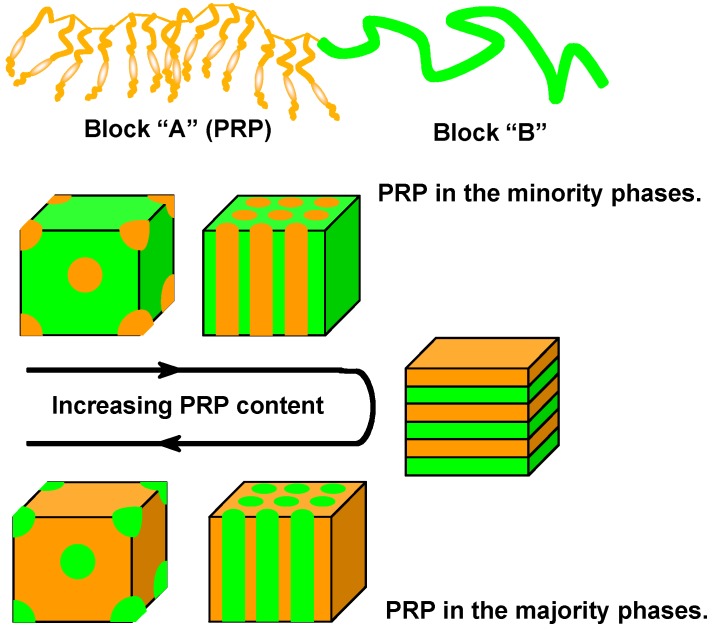
Microphase separation of AB-type PRBCs with well-defined structures.

Because of the immiscibility of a PRP and other blocks of PRBCs, the PRP segment self-assembles into a rich variety of nanostructures, which is quite similar to the common BCs without chromophores [15,16,17,18]. Generally, AB-type diblock copolymers form separated phases (e.g., spheres, cylinders), lamellae or continuous phases by gradually increasing the PRP content in PRBCs, which makes their properties different from those of PRP blends or random copolymers. Figure 1 shows schematic representation of microphase separation of AB-type PRBCs, in which the block “A” and “B” represent a PRP segment and a non-PRP block, respectively.

Although the PRP block constituents the nanoscaled minority phase (<100 nm), its photoresponsive sensitivity is still retained. For instance, when an AZ is used as a chromophore in PRBCs, the AZ in the nanoscale-separated phase can be photo-manipulated into an ordered state having its transition moment almost perpendicular to the polarization direction of the actinic light according to the Weigert effect [19]. 

Confining a PRP block in a separated phase, which is smaller than the wavelength of visible light (400–700 nm), may eliminate the scattering effect and improve the optical performance of PRBCs. When the volume fraction of the PRP block is far larger than that of non-PRP blocks, the PRP segment enables to form the majority phase upon microphase separation. Such kinds of PRBCs behave similarly with that of the PRP. Furthermore, additional performances such as hydrophilicity [12,13,14], crystallization, optical transparency [20,21], thermoplastics [22], ionization [23], water solubility [24], and amphiphilicity [25,26,27] can be acquired by special molecular design of non-PRP blocks. Since the engrossing PRBC offers an effective and convenient chance to design advanced materials by integrating AZs and other chromophores with additional functionalities, it has become one of the emerging topics in photoresponsive macromolecular materials.

## 2. Synthesis of Well-Defined PRBCs

The inherent microphase separation of BCs provides a convenient and economic method to fabricate regular nanostructures by top-down nanotechnology [28,29]. To show regularly ordered nanostructures, BCs should have well-defined structures, and each block of BCs has to be larger than a certain minimum molecular weight. Several polymerization methods, such as anionic, cationic, free radical and metal-catalyzed polymerization have been explored to synthesize PRBCs that meet these requirements. 

### 2.1. Direct Polymerization

**Figure 2:**
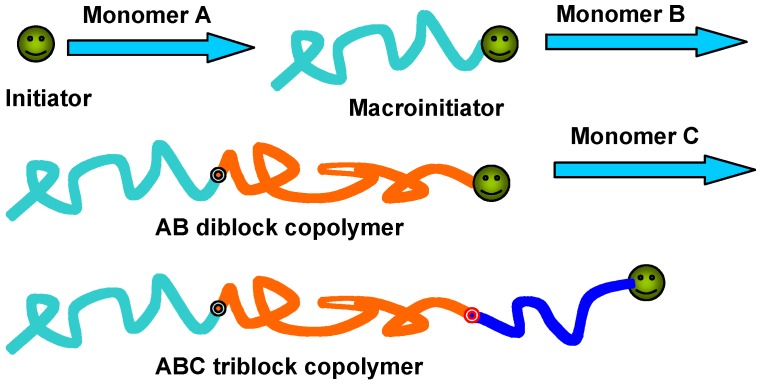
Scheme of preparation of well-defined PRBCs by direction polymerization.

Direct polymerization of chromophore-containing monomers via living processes is one of the most effective ways to synthesize well-defined PRBCs. In this method, a mono-dispersed macroinitiator should be firstly prepared, which is then used as a macroinitiator for the subsequent polymerization of chromophore-containing monomers, as shown in Figure 2. 

Finkelmann *et al*. first synthesized AB-type PRBCs by direct anionic polymerization of an AZ monomer [30]. Polymerization of polystyrene (PS)-based diblock copolymers was carried out from a PS-lithium capped with 1,1-diphenylethylene, while the poly(methyl methacrylate) (PMMA)-based diblock copolymers were prepared by addition of MMA monomers to the “living” AZ polyanion, obtained by reaction of 1,1-diphenyl-3-methylpentyl lithium with an AZ monomer in THF at a lower temperature. By this method, a series of well-defined PRBCs were prepared with controlled molecular weights and narrow polydispersities [30,31].In addition to the living anionic polymerization technique, many commercial polymers and copolymers were synthesized by radical processes. The major success was the large number of monomers could undergo free radical polymerization in a convenient temperature range with the minimal requirement for purification of monomers and solvents. Undoubtedly, atom transfer radical polymerization (ATRP) method is one of the most popular among controlled/living radical polymerization, whose products often possess well-defined structures and narrow molecular-weight polydistributions [32,33]. Since ATRP allows for a control over the chain topology, the composition and the end functionality for a large range of radically polymerizable monomers [32], many PRBCs with specified structures have been synthesized by this approach [12,13,14,34,35,36,37,38,39,40,41,42]. 

**Figure 3:**
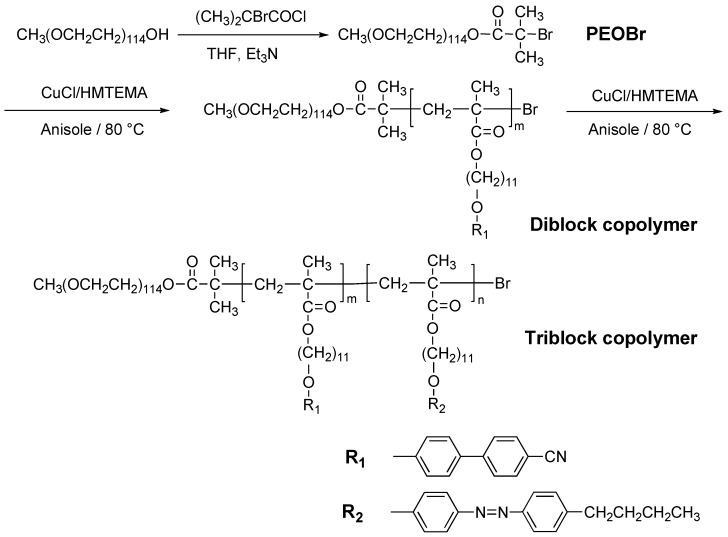
Preparation of amphiphilic PRBCs by a modified ATRP method.

Recently, a modified ATRP method was developed to prepare novel amphiphilic PRBCs consisting of a flexible poly(ethylene oxide) (PEO) as hydrophilic segment and a poly(methacrylate) containing an AZ moiety in side chain as hydrophobic LC segment [14]. The unique characteristic of the obtained PRBCs was the nanoscaled microphase separation, in which a regular array of PEO nanocylinder (10–20 nm) with a periodicity of about 20 nm was dispersed in a smectic matrix. Starting from commercial available PEO, Yu *et al*. applied this approach to prepare several ABC-type PRBCs with photocontrol performances, as shown in Figure 3 [20,21]. 

Besides ATRP, other controlled/living radical polymerization techniques such as reversible addition/fragmentation chain transfer polymerization (RAFT) [43,44] and nitroxide-mediated polymerization (NMP) [45] was also explored to synthesize PRBCs with designed molecular constitution. 

### 2.2. Post Functionalization

**Figure 4:**
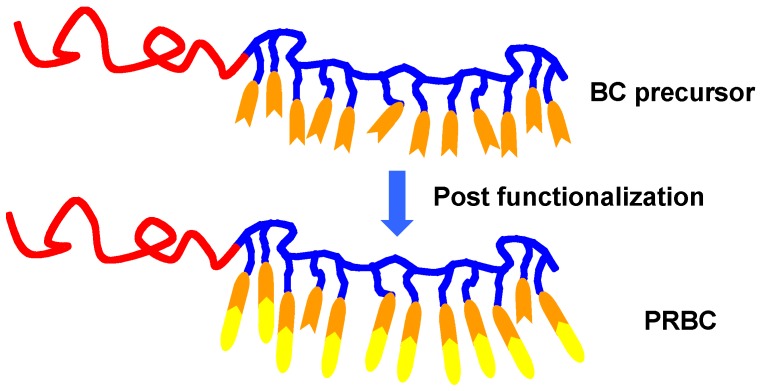
Schematic illustration of PRBCs prepared by post-functionalization.

The PRBCs can also be prepared by post functionalization of active groups in BC precursors, as shown in Figure 4. In 1989, Adams and Gronski first prepared BCs with cholesteryl groups by post-polymerization reaction [46]. Then Ober *et al*. synthesized a family of well-defined PRBCs by a polymer analogue reaction starting from poly(styrene-*b*-isoprene) with a high content of pendent vinyl (and methylvinyl) [47]. Quantitative hydroboration chemistry was used to convert the pendent double bonds of the isoprene block to hydroxyl groups, to which the photoresponsive groups were attached by acid chloride coupling. Due to the many possibilities to functionalize the hydroxyl group in poly(2-hydroxyethyl methacrylate), it was used to prepare PRBCs by other groups using a similar polymer analogue way [48,49].

Similar to the post-esterification method, a post azo-coupling reaction (PACR) was utilized to design AZ-containing PRBCs [50]. Three kinds of AZ chromophores have been summarized by Natansohn and Kumar [1,6]. The first “AZ” carries relatively poor π–π* and n–π* transition overlap and the lifetime of the *cis*-isomer is relatively long. The second one is “amino-AZ” having significant overlap of the two bands and the *cis*-isomer lifetime is shorter. The third AZ is “pseudostilbene”, where the AZ is usually substituted with electron-donor/acceptor substituents. It is difficult to directly synthesize PRBCs containing amino-AZ or their push-pull derivatives by ATRP because of the inhibition effect of the amino-AZ-containing monomers toward free radicals [50]. PACR was a convenient way to introduce such an AZ into macromolecular chains [51]. Wang *et al*. prepared amorphous AZ-containing PRBCs by the PACR of an amphiphilic PEO-based precursor and then studied the photoinduced shape change of the spherical aggregates formed by the post-functionalized PRBCs [52]. Recently, a post Sonogashira cross-coupling reaction of a reactive polymer precursor was used to prepare highly birefringent PRPs. This seemed to be one of candidate reactions to synthesized well-defined PRBCs [53].

### 2.3. Supramolecular Self-Assembly 

Upon supramolecular self-assembly, low-molecular-weight additives can be used to adjust the properties of BCs by hydrogen bonding. Ikkala *et al*. first introduced this concept in a PS-*b*-poly(4-vinylpyridine) (P4VP), which was stoichiometrically complexed with pentadecylphenol molecules to form supramolecular complexes [54]. Then, Stamm *et al*. fabricated well-ordered nanostructures by supramolecular assembly of PS-*b*-P4VP and 2-(4-hydroxyazobenzene) benzoic acid (HABA), consisting of nanocylinders formed by P4VP–HABA associates in the PS matrix [55]. Extraction of HABA with a selective solvent resulted in nanochannel membranes with a hexagonal lattice of hollow channels in the nanocylinders crossing the membrane from the top to the bottom. In addition, supramolecular self-assembly between PS-*b*-poly(acrylic acid) (PAA) and imidazole-terminated hydrogen-bonding mesogenic groups was also used to prepare PRBCs [56]. Owing to the attached LC properties, the nanostructures in the obtained PRBC could be oriented by using an alternating-current (AC) electric field, in a direction parallel to the electrodes.

### 2.4. Special Reactions

**Figure 5:**
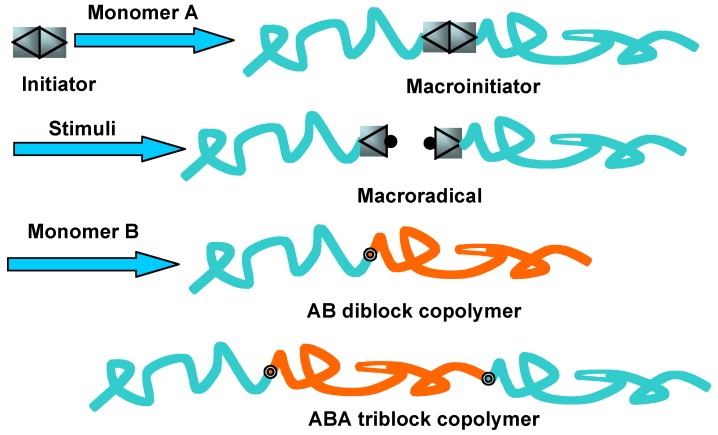
Scheme of PRBCs prepared from a special reaction.

Some special reactions with a particularly designed route have been used to synthesize PRBCs, and such a reaction route includes at least two polymerization processes. As shown in Figure 5, an initiator with a specified structure is used to prepare a macroinitiator with only one initiator in one polymer chain. Under certain conditions like light irradiation or thermal treatment, macroradicals can be formed by the decomposition of the macroinitiator. This induced additional radical polymerization of a chromophore-containing monomer from the decomposed points in the segment. Thus, AB-type or ABA-type PRBCs can be obtained by termination of the macroradicals. For instance, a series of poly(vinyl ether)-based PRBCs were synthesized by using living cationic polymerization and free-radical polymerization techniques [57]. 4.4′-Azobis(4-cyano pentanol) (ACP) was used to quantitatively couple two well-defined polymers of living poly(vinyl ether), initiated by the methyl trifluoromethane sulfonate/tetrahydrothiophene system. Then, the ACP in the main chain was thermally decomposed to produce polymeric radicals, which was then used to initiate the polymerization of MMA or styrene to obtain PMMA-based or PS-based PRBCs (AB or ABA types). Although the obtained PRBCs showed narrow polydistributions, no ABC-type PRBCs were prepared by such a reaction route. 

## 3. Properties of PRBCs

### 3.1. Photoresponsive Behaviors

Being one of multi-functional polymeric materials, PRBCs combine the photochemical properties of PRPs and microphase separation of well-defined BCs. On the one hand, the PRP block and non-PRP segments cannot be completely miscible, unlike statistically random copolymers in which the chromophores are homogeneously dispersed over the whole bulk films. On the other hand, the PRBCs cannot form macroscopically phase-separated structures like polymer blends, which is attributed to the tightly covalent connection between the PRP segment and non-PRP blocks. 

**Figure 6:**
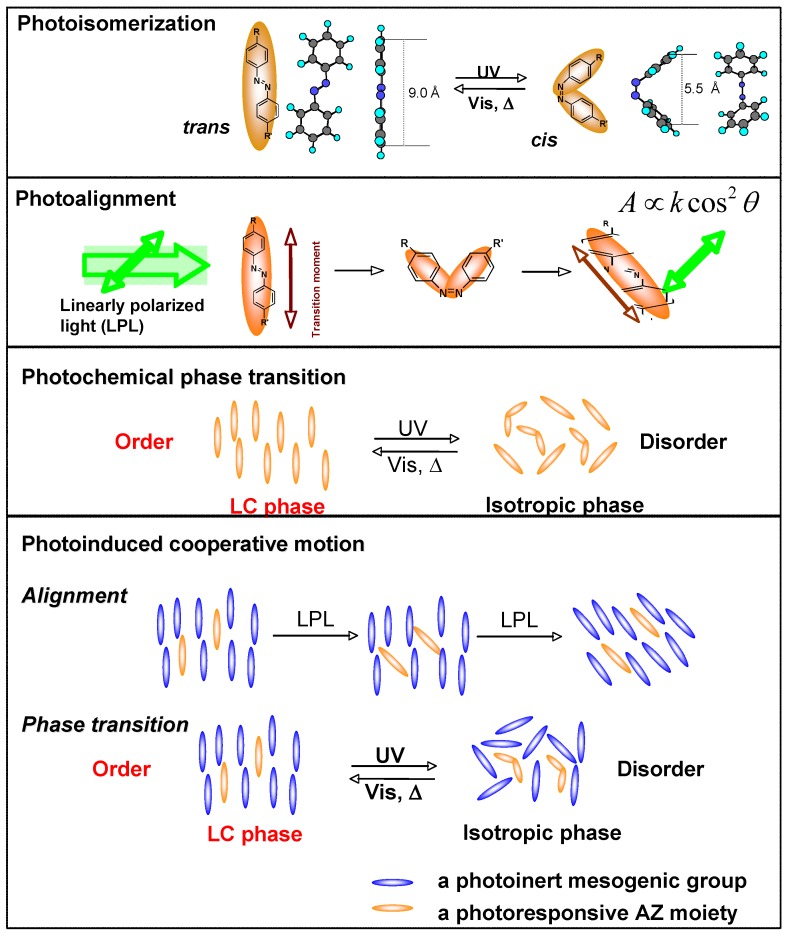
Properties of AZ-containing PRBCs inherited from AZ homopolymers. A is the absorption of AZs, θ represents the angle between the polarization direction of the linearly polarized light and the transition moment of an AZ moiety.

An AZ moiety is well-known for its reversible photoisomerization, and it may acts as both a mesogen and a photoresponsive moiety when it is attached to a polymer by a soft spacer. Both photoisomerization and photoinduced LC-to-isotropic phase transition are involved in microphase separation in the LC PRBC due to the immiscibility between AZ-containing PRP block and the non-AZ segments. Therefore, the microphase separation of PRBCs might be influenced by photoresponsive features of the PRP block, and vice versa. It was known that the self-organized nanostructures upon microphase separation could have effect on the photoresponsive behaviors of the PRP blocks, [37]. The photocontrol and supramolecular self-assembly make AZ-containing PRBCs superior to that of homopolymers or random copolymers. Generally, AZ-containing PRBCs inherit most of the excellent properties of AZ homopolymers [1,2,3,4,5,6,7,8,9,10,11], such as photoisomerization, photoalignment and photochemical phase transition, since the trans-AZ could be a mesogen because of its rod-like molecular shape, whereas the cis-AZ never shows any LC phase due to its bent shape [7,58]. All the illustrations of the photoresponsive performances are shown in Figure 6. 

**Figure 7:**
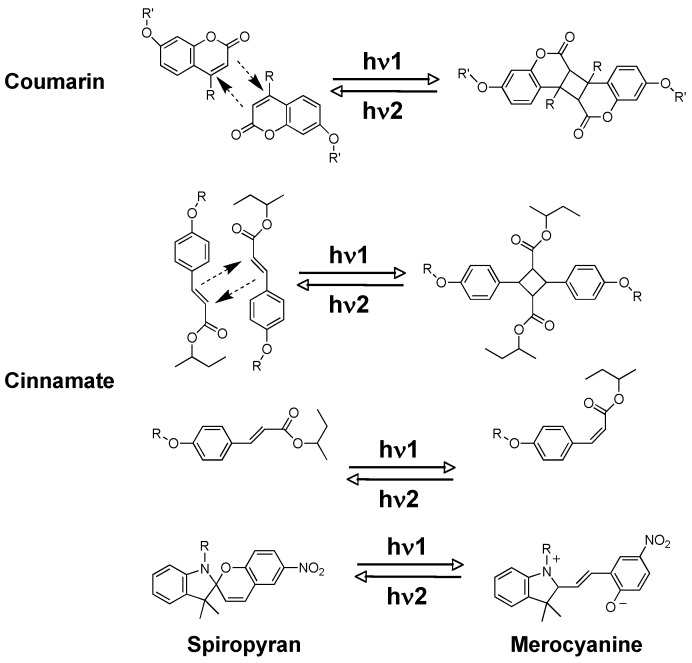
Photoresponsive properties of coumarin, cinnamate and spiropyran derivatives.

Apart from AZs, other chromophores such as cinnamates and coumarins are usually used as photoresponsive groups in preparation of PRPs. Upon photoirradiation, coumarins show a [2+2] dimerization reaction to form cycobutane derivatives, which can be subsequently photocleaved by choosing an actinic light with a suitable wavelength (Figure 7) [59]. Differently, both dimerization and isomerization could be reversibly induced in cinnamate-containing PRPs upon light irradiation [60,61]. With a different photoreaction mechanism, the photoresponsive behavior of spiropyran-containing PRPs arises from a photochemical 6π electron ring-closure reaction, leading to a photocontrolled reversible change between a colorless closed spirostructure and a colored open merocyanine structure.

Because of the well-defined structure, PRBCs show excellent and different features from random copolymers. Recently, the molecular cooperative motion (MCM) between photoresponsive AZ moieties and other photoinert groups was studied in triblock copolymers with specifically designed structures [20,39,44]. Triggered by linearly polarized laser, AZs were first photoaligned due to the Weigert effect [19], and then non-photoactive groups were oriented together with the aligned AZ moieties under the function of MCM, although they did not absorb the actinic light [62,63]. 

**Figure 8:**
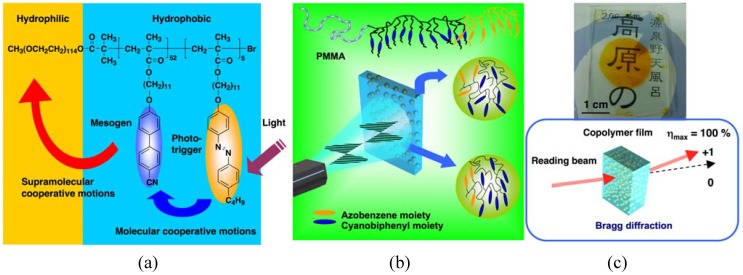
Photoinduced MCM and SMCM of PRBCs. A PRP forms the majority phase (a) and the minority phase (b), respectively. (c) A photograph of a PRBC film with 200 μm thickness (left) and scheme of the recorded Bragg grating (right).

When AZs and photoinert mesogens formed the majority phase (a continuous matrix), the photocontrolled orientation was transferred to the microphase-separated nanodomains inside them owing to SMCM (Figure 8) [39]. Interestingly, the photoinduced MCM was confined in nanoscaled regions by microphase separation, when AZs and other mesogenic blocks were situated in the minority phases (separated phases) [20]. No obvious influence on the substrate was observed, since the photoalignment occurred only in the incontinuous phase, and then the phototriggered orientation was disrupted by the glassy substrates. Owing to the nanoscaled MCM, the scattering of visible light was avoided by confining the mesogenic domain to the nanoscale, which improved the optical transparency of PRBCs. Accordingly, thick films (>100 μm) with a high transparence and a low absorption based on the microphase separation of PRBCs were obtained, enabling them to record Bragg-type gratings for volume storage. Figure 8c gives the possible scheme of Bragg diffraction based on transparent thick films (about 200 μm) of an ABC-type PRBC. Furthermore, the stability of photoinduced orientation was greatly improved by decreasing the photoresponsive AZ content in PRBC composition.

### 3.2. Non-Photoresponsive Properties 

**Figure 9:**
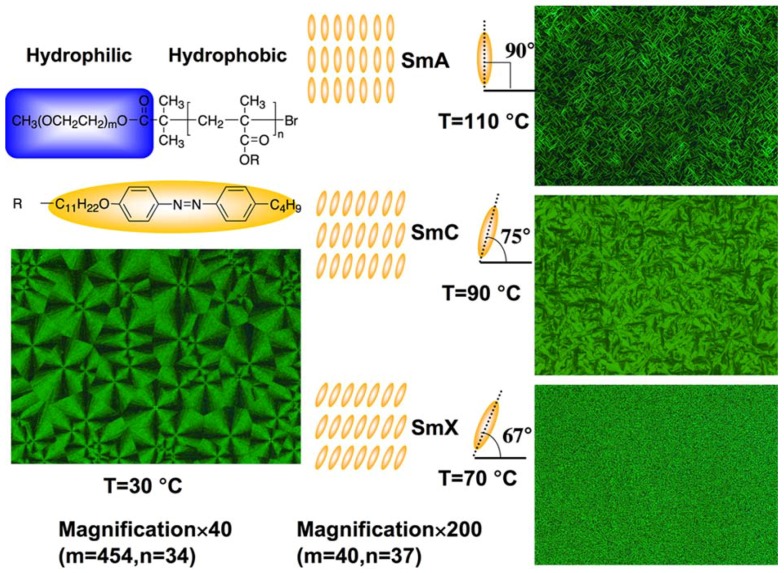
POM images and possible smectic LC layer structures of PEO-based PRBCs.

Being constituent parts of well-defined PRBCs, non-PRP blocks also influence the properties of PRBCs. For instance, introduction of PEO as one block endowed PRBCs with hydrophilicity, ion conductivity and crystallization [14,64,65,66]. In Figure 9, both PEO crystals and LC phases were clearly observed with a polarizing optical microscope (POM). When the repeated unit of PEO was far higher than that of the PRP block, typical spherulite structures with birefringence were observed. The process of spherulization generally started on a nucleation site and continues to extend radially outwards until a neighboring spherulite was reached. This led to the spherical shape of the spherulite. When the PRP block formed the majority phases, LC textures of focal conic fan were obtained at a higher temperature, indicating smectic LC phases. In Figure 9, further experimental results of wide-angle X-ray power diffraction (WXRD) indicated that the LC phase of smectic A, C and X was obtained at different temperatures, corresponding to the LC textures in POM images, respectively [14].

Introduction of poly(2–(dimethylamino)ethyl methacrylate) p(DMAEMA) brought water solubility to PRBCs, when the hydrophobic PRP segment was short enough [24]. Using PMMA as one block, the PRBC films showed good optical transparency [20,21,35,67]. The PS block provided the designed PRBC with a high glass-transition temperature (Tg) [38], and offered confinement effects on the photoalignment of LC side chains [36]. Putting rubbery poly(*n*-butyl acrylate) as middle block in ABA-type triblock copolymers, thermoplastic elastomers were obtained [22], which contrasted with conventional thermoplastic elastomers. It was also reported that semi-fluorinated alkyl substituents in AZs decreased the surface energy of PRBC films [68]. In addition, poly(2-methoxyethyl vinyl ether)-based PRBCs supplied the polymer surface with the thermally-controlled water wettability [69].

### 3.3. Microphase Separation of PRBCs

PRBCs are one fascinating class of soft materials, showing a rich variety of microphase-separated nanostructures. As shown in Figure 1, the morphologies can be controlled to be spheres, cylinders, or lamellae, depending on the length, chemical nature, architecture and repeated units in each block [15,16]. When the PRP segment showed LC properties, the inherent mesogenic ordering might effect on the thermodynamic process of microphase separation by SMCM. The investigation of PRBCs with LC performances undoubtedly fertilize the detailed illustration of microphase separation, and novel nanostructures and phase behaviors are expected by the supramolecular self-assembly. 

**Figure 10:**
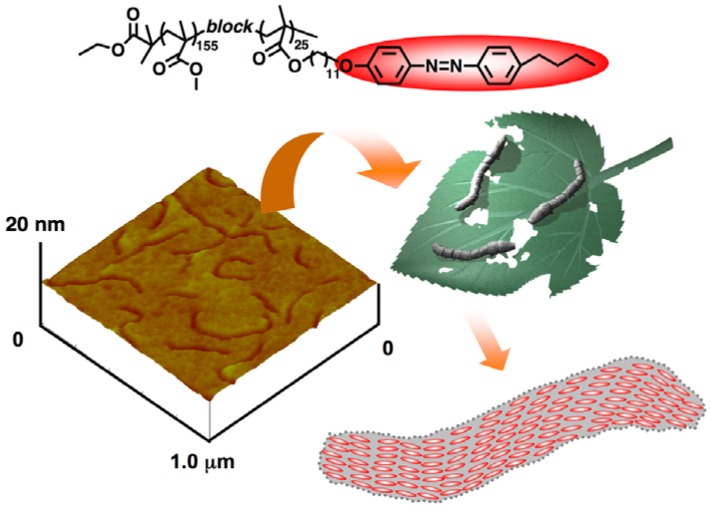
Wormlike nanostructures obtained in a PRBC showing an LC phase.

Recently, a novel wormlike nanostructure composed of photoresponsive AZ mesogens was observed in PMMA_155_-*b*-PMA(11Az)_25_ (Figure 10). Due to the difference in elastic modulus between amorphous PMMA and the LC phases, wormlike microphase-separated domains were clearly observed in atomic force microscopic (AFM) images [21]. According to the component of the PRBC, the wormlike domain with a width of 25 nm should be self-segregated by the PRP blocks, leading to the disappearance of an LC texture after microphase separation because of the limited POM resolution. The wormlike nanostructure might be caused by the balance between microphase separation and LC self-assembly, which makes it different from the phase-segregated morphologies of BCs with weak molecular interactions [21].

**Figure 11:**
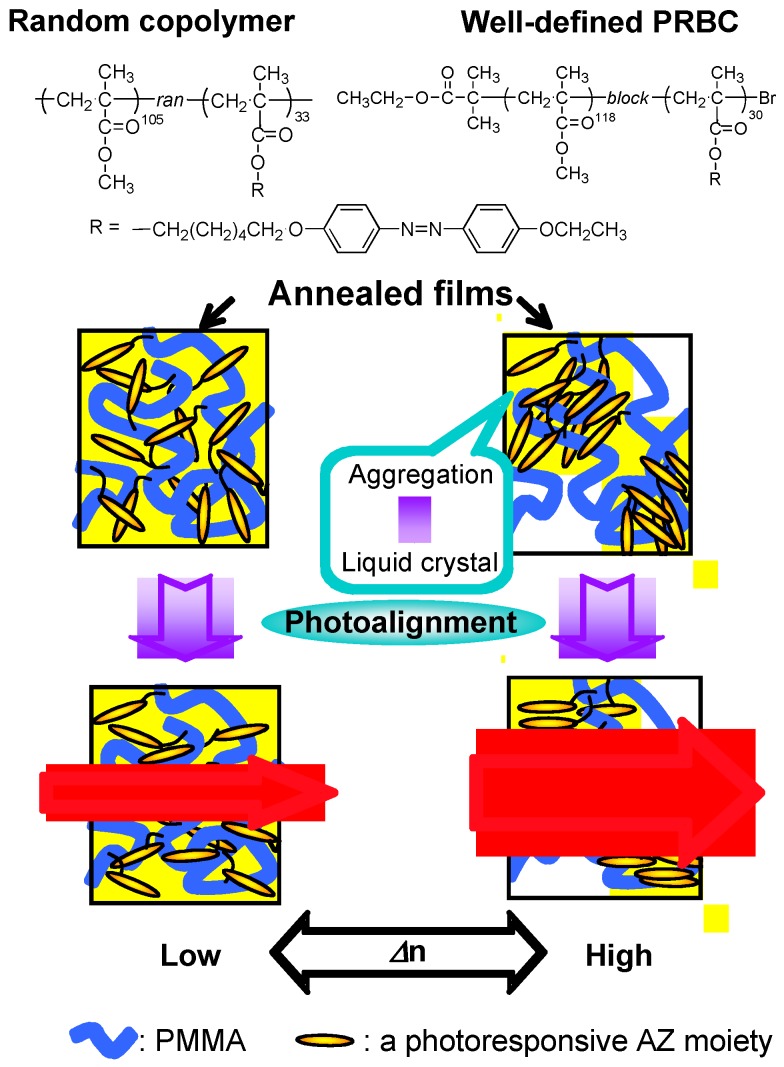
Schematic illustration of a well-defined PRBC and a random copolymer with a similar chromophore content (about 22 mol %).

Upon microphase separation, the PRP block with a relative lower content aggregates together, enabling them to easily show an LC order in local areas, whereas a random copolymer with a similar AZ content shows a statistically molecular structure, in which the chromophore is dispersed homogeneously within the matrix of non-PRP blocks. Since the photoresponsive group and other copolymer segments were completely miscible resulting in an amorphous phase, no microphase separation can be observed [70,71]. Therefore, PRBCs showed LC phases more easily than a statically random copolymer with a similar low content of photoresponsive mesogens [71]. Although the random copolymer had a similar AZ composition of about 22 mol % as that of the well-defined PRBC (Figure 11), no LC phase was observed. The acquired LC property might endow the PRBC with advanced performances such as physical anisotropy, self-organization, long-range ordering, MCM and SMCM. Since the segregated PRP block was incompatible with the PMMA segments, a cooperative effect occurred in the process of photoalignment, leading to a larger photoinduced birefringence than in the random copolymer. Besides, H- or J- aggregates were easily formed, since the PRP block exhibited a high local concentration in the microphase-separated domains [14,36,71].

The microphase-separated morphologies also influence the LC properties of the PRP blocks [36,47]. For instance, a PS-based LC PRBC with mesogenic nanocylinders embedded within the PS matrix exhibited a clearing point, 22 °C higher than that of the LC PRBC with lamellar structures, even though the former sample has a slightly lower molecular-weight (Mn) than the latter one. Ober *et al*. proposed that the nanocylindrical structures in PRBCs might stabilize the smectic mesophase within it than the lamellar morphology [47].

## 4. Control of Microphase Separation of PRBCs

The microphase separation is one of the most importation properties of well-defined PRBCs. Classical BCs segregate into nanoscaled phase-domains with periodic structures [72]. The driving force for microphase separation is to achieve the required balance of minimizing the interfacial energy and maximizing the conformational entropy of BCs. However, thin films of microphase-separated BCs typically have less long-range ordering, which limits their further application. Generally, LC polymers and BCs are two types of ordered non-crystalline materials that can undergo self-assembly [73,74]. 

**Figure 12:**
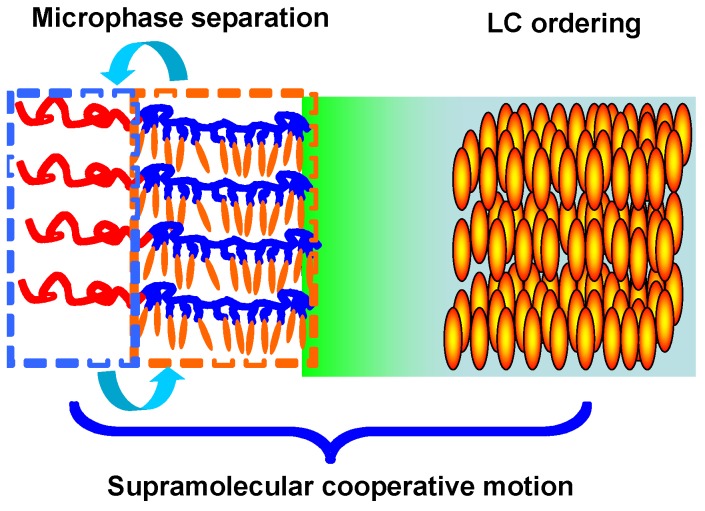
Schematic illustration of supramolecular cooperative motion in LC PRBCs.

From the viewpoint of molecular design, LC PRBCs integrate the unique characteristics of both materials into one single system, which also possesses photoresponsive properties. Such a SMCM was regarded as one of the most effective approaches to control nanostructures in LC PRBCs [12,13], as shown in Figure 12. In the following part, several newly developed approaches to control nanostructures in PRBCs are discussed.

### 4.1. Thermal Annealing

Formation of large-area periodic nanoscale structures using supramolecular self-organization is of great interest because of the simplicity and low cost of the fabrication process [75]. Although macroscopically ordered microphase separation was successfully prepared in common BCs [18,29], both high reproducibility and mass production of such regularly ordered nanostructures through self-assembling processes still remain challenging. 

**Figure 13:**
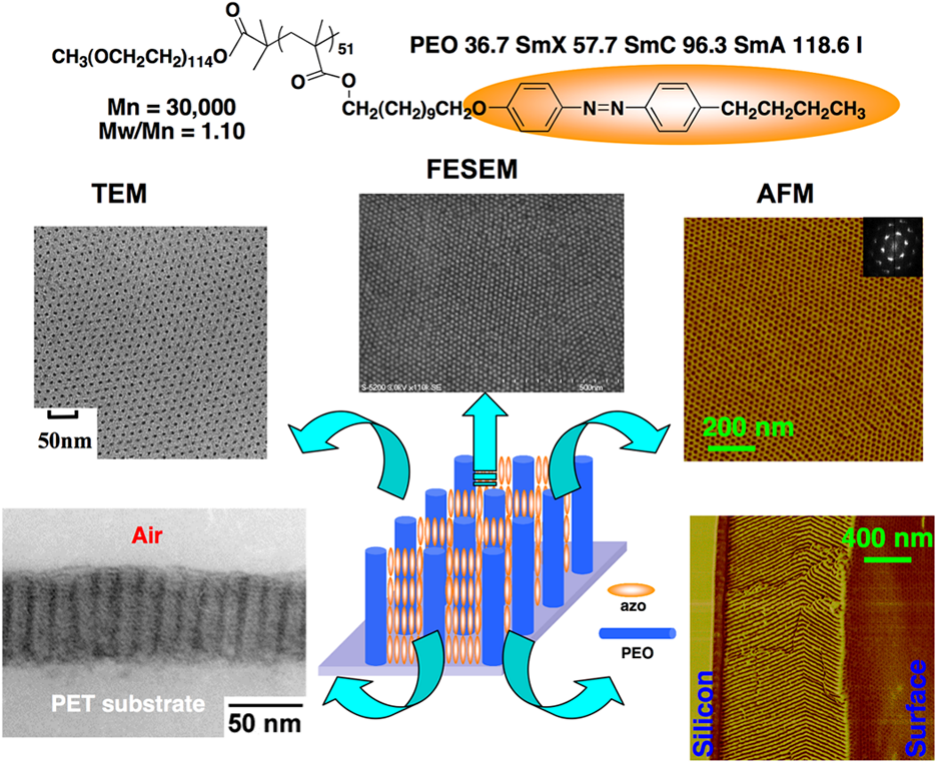
Perpendicular array of nanocylinders and photoresponsive mesogens in a PRBC film by thermal annealing.

Specially designed amphiphilic LC PRBCs are good candidates to produce low cost materials with self-assembled nanostructures, leading to industrial applications in future engineering plastics. As shown in Figure 13, all the TEM, AFM and FESEM pictures reveal beautiful PEO nanocylinder structures with hexagonal packing, in an alignment direction perpendicular to the substrates [14,76,77]. 

Although no external driving forces were exerted on the PRBC, the regular periodic arrangements of nanocylinders were not limited to the film surface, and cross-sectional images confirmed the formation of three-dimensional (3D) arrays. It was proven that the 3D arranged nanostructures should be assisted by the out-of-plane orientation of the photoresponsive mesogens, which formed the continuous phase of the PRBC [14,76]. In the cross-sectional TEM image, the smectic layer structures in the PRP block were observed normally to the PEO nanocylinders (parallel to the substrate). This was also confirmed by using small-angle X-ray scattering (SAXS) measurements [77]. Such cooperative effect between PEO nanocylinders and the mesogenic orientation was a result of SMCM at a temperature higher than the clearing point, which decreased the viscosity of PRBCs, and enabled the interaction between the microphase separation with the smectic LC ordering to proceed completely. On the other hand, the film thickness and the substrate properties exert a great influence on the nanoscaled morphologies formed by microphase separation of PRBCs. By careful control of the experimental conditions, well-ordered arrays of nanoscaled phase domains can be extended to a macroscopic scale, as analogous to “a polymeric single crystal”. More interestingly, such dotted patterning of PEO nanocylinders was achieved over several centimeters [77].

### 4.2. Mechanical Rubbing

Following the SMCM in LC PRBCs (Figure 12), a long-range order of PEO nanocylinders can be fabricated along the direction of LC alignment, suggesting the application of LC alignment techniques to control microphase-separated nanostructures in PRBCs. Although there are several LC alignment methods such as rubbing, optical, electric or magnetic field, Langmuir-Blodgett films, silicon-oxide treated surface, and oblique evaporation, only the rubbed method is widely utilized in commercial production of LC displays (LCDs).

**Figure 14:**
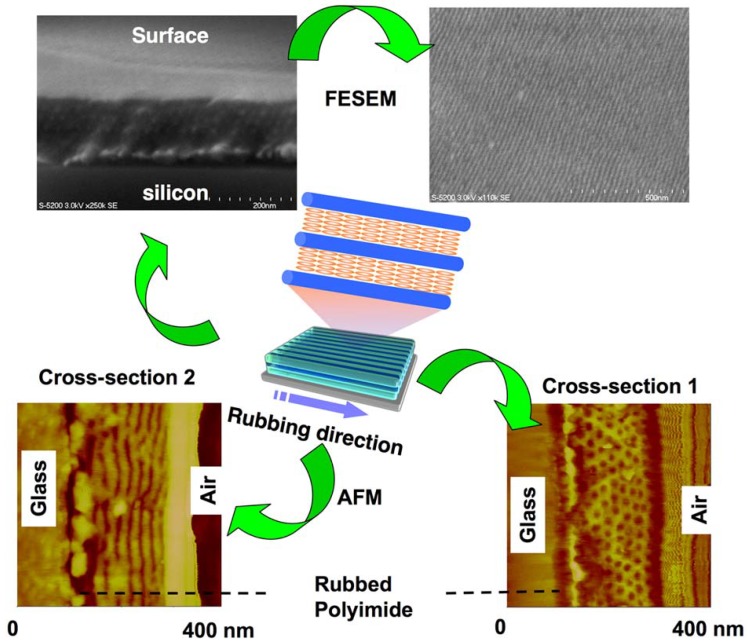
Fabrication of a parallel nanocylinder array in a PEO-based PRBC by the mechanical rubbing method.

Parallel processes for patterning densely-packed nanostructures are often required in diverse areas of nanotechnology [78]. The regular nanostructures by self-assembly in amphiphilic PRBCs provided unique opportunity to prepare such a parallel stripe pattern by SMCM. To achieve this, a rubbing technique was chosen to homogeneously align LCs in PRBCs. The periodic ordering of oriented mesogens might be transferred to nanocylinders formed with non-PRP blocks by SMCM. It is well-known that LC molecules can be aligned along the rubbing direction on rubbed polyimide surfaces because of the lower energy level along the rubbing-induced grooves [79,80,81]. Such a method exhibited strong influence on the LC PRBC, as shown in Figure 14. After rubbing treatments, a parallel array of PEO nanocylinders coinciding with the LC alignment was clearly observed by AFM and FESEM images. This defect-free nanocylinder array was acquired over arbitrarily large areas on the surface of rubbed polyimide films. Similar to other methods of controlling microphase segregation in amorphous BCs, such as electric field, crystallization, controlled interfacial interaction, chemically or topologically patterned substrates, the rubbing technique exerted a forceful function on 3D arrays of nanostructures, which was suggested by the cross-sectional images of AFM and FESEM (Figure 14). The rubbing treated samples showed a regular surface relief of about 3 nm with a periodicity of 24 nm, possibly due to the confined crystallization of the PEO blocks embedded in the ordered LC phases [82]. Obviously, both the nanocylinder dimension and the surface periodicity could be easily adjusted by the volume fraction of PEO or PRP blocks in the PRBCs. The macroscopic 3D nanocylinder array with a long-range order was successfully achieved with arbitrarily controlled direction in plane, in which a novel pathway of controlling nanoscopic domains was opened over large areas [12,13]. The rubbing method has advantages of simplicity, convenience and low cost over other approaches, which can create opportunities for manufacturing nanoscopic devices. In the processes of nanocylinder alignment, the rubbing acts on the microphase domains by a bridge of LC media, implying that the nanostructures can be controlled by way of modulating LC alignment, which technologically implicate that other LC manipulating methods can be used to regulate microphase separation of PRBCs.

### 4.3. Photoalignment

In the mechanical rubbing process to control LC alignment, dust or static electricity produced can introduce defects in an ordered array of microphase-separated nanostructures of PRBCs. Moreover, this method can only be applied for a flat surface. Therefore, noncontact methods, such as photocontrol approaches were explored [12], because light is one of the most convenient and cheap energy, whose intensity, wavelength, polarization direction as well as interference patterns also can be manipulated simply. Simplified fabrication of microphase-separated nanostructures in PRBCs is expected by photocontrol. 

**Figure 15:**
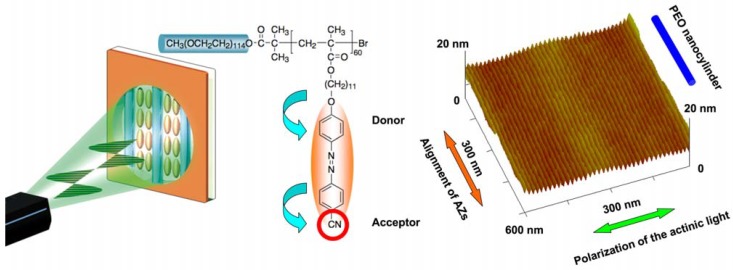
Photoalignment of microphase-separated nanocylinders in an LC PRBC.

Upon irradiation with linearly polarized light, AZs are known to undergo photoalignment with their transition moments almost perpendicular to the polarization direction [19]. Such ordering can be directly transferred to non-photoresponsive mesogens by MCM coinciding with the ordered AZ moieties. By incorporating the photoalignment of AZs into PRBCs with SMCM, the molecular ordering of AZs can be transferred to a supramolecular level, leading to well-ordered nanostructures with photocontrollability [83]. In Figure 15, a linearly polarized laser beam was used to control PEO nanocylinders self-assembled in an amphiphilic PRBC with a smectic LC phase. Upon annealing without photoirradiation, hexagonal packing of the nanocylinders perpendicular to the substrate was obtained due to the out-of-plane orientation of the mesogens (Figure 13). Then photoalignment of AZs was carried out at room temperature, and then the anisotropic PRBC films were thermally annealed at a temperature just lower than the LC-to-isotropic phase-transition temperature. A parallel array of nanocylinders was achieved in an aligned direction perpendicular to the laser beam. 

Recently, Seki *et al*. reported control of microphase-separated nanocylinders by periodic change in film thickness induced by mass transfer in recording SRGs [84]. In their preparation, the film thickness was strictly modulated and the PRBC was mixed with a low-molecular-weight LC (5CB) to photoinduce a large mass transfer. 5CB was then eliminated after grating formation. To simplify the process, they adopted a polarized beam to control the nanocylinders in PS-based PRBC films [38]. Defects appeared in the microphase-separated nanostructures, probably caused by incomplete microphase separation resulting from a high Tg of the PS block. 

In non-doped films of LC PRBCs, macroscopically parallel patterning of PEO nanocylinders can be obtained easily in an arbitrary area by the photocontrol. Furthermore, the noncontact method might provide the opportunity to control nanostructures even on curved surfaces. Based on the SMCM, the orientation of microphase-separated nanocylinders dispersed in photoresponsive matrixes should agree with the LC alignment, which is expected to provide complicated templates for top-down-type nanofabrications such as lithography and beam processing.

### 4.4. Electric and Magnetic Fields

**Figure 16:**
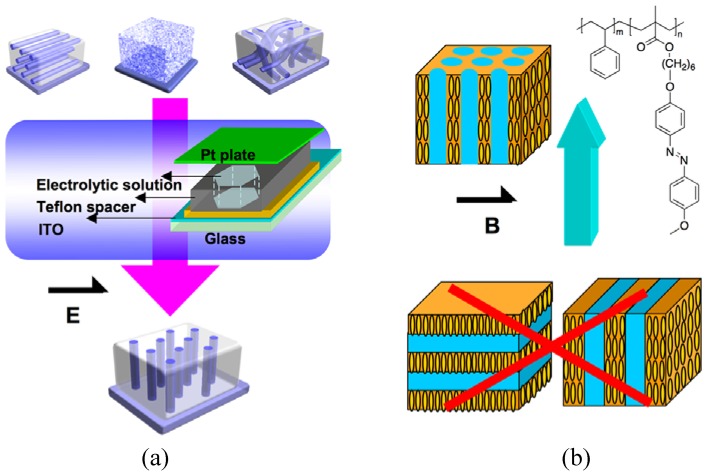
Control of nanocylinders in PRBCs by electric (a) and magnetic fields (b).

More recently, Kamata *et al*. developed an electrochemical method to control alignment of PEO nanocylinders perpendicular to the substrate in PEO-based PRBC films [85]. As show in Figure 16a, the PRBC films were prepared by spin coating on ITO glass kept at 50 °C for 2 days. Using the ITO glass as a working electrode, a sandwich-type cell was assembled with a Teflon spacer and an injected KBr aqueous solution as electrolyte. Under the function of an electrolytic potential in the potentiostatic mode with Pt counter electrode and Ag/AgCl reference electrode, all the nanocylinders were oriented parallel to the electrolytic field as the lowest energy alignment, in spite of the microphase-separated state, parallel or random alignment of PEO nanocylinders. It was believed that ion diffusion locally induced in the vicinity of the electrode could allow the hydrophilic nanocylinders normal to the substrate, making it possible to manipulate the microphase-segregated microdomains [85].

For hydrogen-bonded PRBCs, an AC electric field was used to rapidly align the nanostructures at temperatures below the order-disorder transition but above *T*_g_ [56]. The low-molecular-weight mesogens played an important role in controlling microphase separation. The fast orientation switching of the nanostructures was attributed to the dissociation of hydrogen bonds, which might be used to control nanostructures in supramolecular PRBCs. 

Similar to the electric field, the magnetic field was used to manipulate the microphase separation in PRBCs. The noncontact orientation method provides a higher degree of freedom for sample shapes than the mechanical orientation method. Furthermore, no danger presents, such as the dielectric breakdown that can be encountered in the electrical orientation approach. The uniform orientation of LCs could be obtained over the whole region of a sample, regardless of the macroscopic shape of the sample and the strength of the magnetic field [86]. In Figure 16b, hexagonally packed nanocylinders dispersed in mesogenic matrixes were aligned along the magnetic field upon annealing for a longer time (> 2h) at a nematic LC phase. But the magnetic field showed no function on the lamellar-nanostructured PRBC, possibly because ordered lamellar microdomains with a long correlation length were only rearranged very little [87]. Although the LC was magnetically aligned in nanoscale layers, it showed no influence on the inverse continuous phase (Figure 16b) [88].

### 4.5. Other Methods

The orientation of both lamellar and cylindrical microdomains in PRBCs can also be obtained with a shearing flow, yielding highly oriented samples [89,90,91,92]. During oscillatory shearing of PS-based PRBCs in LC or isotropic phases, the LC phase showed a distinct effect on the orientation of microphase separation in PRBCs. Upon the LC-to-isotropic phase transition, the mesogenic orientation was lost, whereas the orientation of nanocylinders was sustained. Interestingly, the uniaxial planar orientation of the mesogens was recovered completely on cooling from an isotropic melt. Such spontaneous reorientation of the LC phase was carried out after repeating the heating and cooling cycles above and below the phase-transition temperature, showing that the oriented nanocylinders acted as an anchoring substrate for mesogens [91,92]. Undoubtedly, other approaches for controlling microphase separation of PRBCs, such as solvent evaporation, film thickness, modified substrates, mixture with homopolymers in addition to roll casting are also expected to be used in PRBCs.

## 5. Applications

### 5.1. Holographic Gratings and Storage

#### 5.1.1. Enhancement of Surface Relief

**Figure 17:**
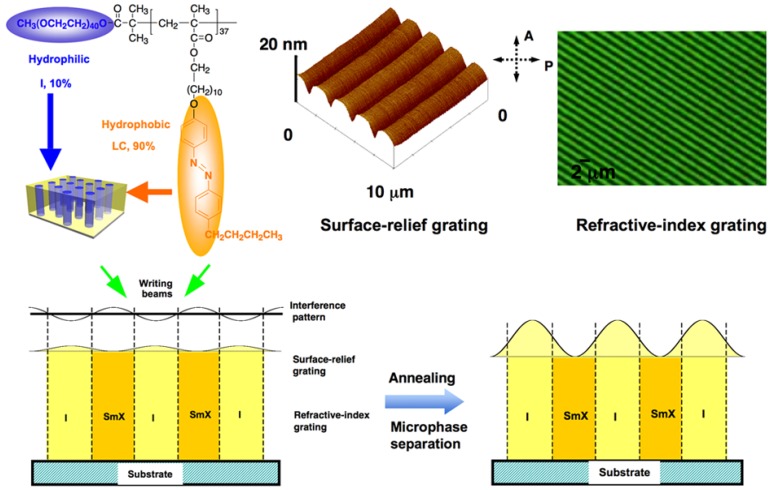
Holographic gratings recorded in PRBCs and enhancement of surface relief upon microphase separation.

Holographic gratings have potential applications in information technology, which have been recorded on PRP films by utilizing their photoresponsive properties [93,94]. The diffraction efficiency (DE) is one of the most important parameters of holographic gratings. In amorphous PRP materials, a surface-relief grating contributes mainly to DE. It was reported that PRBCs were good candidates to control DE by enhancement of surface relief upon microphase separation [95,96,97]. In Figure 17, both a SRG and a refractive-index grating (RIG) were recorded upon irradiation of an interference pattern, in which selective photoisomerization and the isotropic-to-LC phase transition were induced in the bright areas. The DE of the gratings depended strongly on the polarization of the reading beam because of the photoalignment of mesogens.

After grating formation, the SRG structure was clearly observed from the AFM images (Figure 17), in which a sinusoidal curve was obtained. The fringe spacing of the SRG was 2.0 μm, identical to that of the RIG. Then nanoscaled microphase separation was induced by annealing the grating samples, as a result, the surface relief was increased to about 110 nm (18.3% of the film thickness), almost one order of magnitude larger than that before annealing. The peak-to-valley contrast became more explicit after annealing, due to the enhancement of the surface modulation. Furthermore, the sinusoidal shape of the surface profile became a little irregular, indicating that the LC alignment was disturbed upon microphase separation. Together with the enhancement of SRG, the DE increased to about 9.0%, almost two orders of magnitude larger than the DE before annealing. This increased DE may be ascribed mainly to the enhancement of surface modulation.Comparing to other methods to control DE, such as gain effects, mechanical stretch, electrical switch, self-assembly, mixture with LC and cross-linking, the microphase-separation method had advantages of being simple and convenient [95,96]. To precisely control DE of recorded gratings in amphiphilic PRBCs, the effect of recording time on grating formation and enhancement was studied systematically. The best enhancement effect was obtained at 10 s recording upon microphase separation. By adjusting the recording time, the DE was finely controlled from 0.13% to 10% [96]. 

#### 5.1.2. Enhancement of Refractive-Index Modulation 

**Figure 18:**
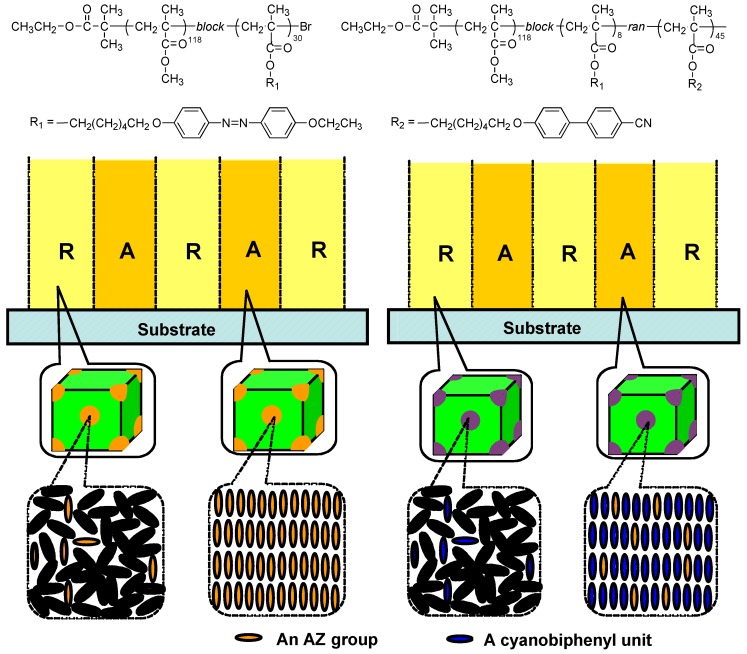
Enhancement of surface-index modulation in PRBCs with photoresponsive groups in the minority phase. (A, aligned, R, random).

By the cooperative effect between photoresponsive AZ moieties and photoinert groups, a small external stimulus can induce a large change in refractive index of the materials, which has been widely used in holographic recording. This is especially useful in AZ-containing BCs with mesogens in the minority phase dispersed in glassy substrates. Then the photoinduced mass transfer was greatly prohibited due to the microphase separation in grating recording, lack of surface-relief structures was observed [67,99] Thus, refractive-index modulation plays an important role in the grating formation in such PRBCs. As shown in Figure 18, holographic gratings were recorded in films of two PMMA-based PRBCs. One was a well-defined AZ-containing diblock copolymer, and the other sample was a diblock random copolymer. Here, the diblock random copolymer consisted of two blocks, in which one segment was PMMA and the other mesogenic block was statistically random. After grating formation, both films showed no SRG, and only RIGs were obtained. Upon irradiation of two coherent laser beams, RIG in the AZ-containing diblock copolymer was recorded by photoalignment of AZs dispersed in phase-separated domains. In contrast, the photoalignment of the AZ was amplified by the photoinert cyanobiphenyl moieties as a result of the cooperative effect in the diblock random copolymer. This led to a similar refractive-index modulation, although the AZ content was lower in the diblock random copolymer. The cooperative motion was confined within the nanoscale phase domains, unlike the case of random copolymers with statistically molecular structures [62,63]. 

#### 5.1.3. Adjusting Fringe Spacing of Gratings 

**Figure 19:**
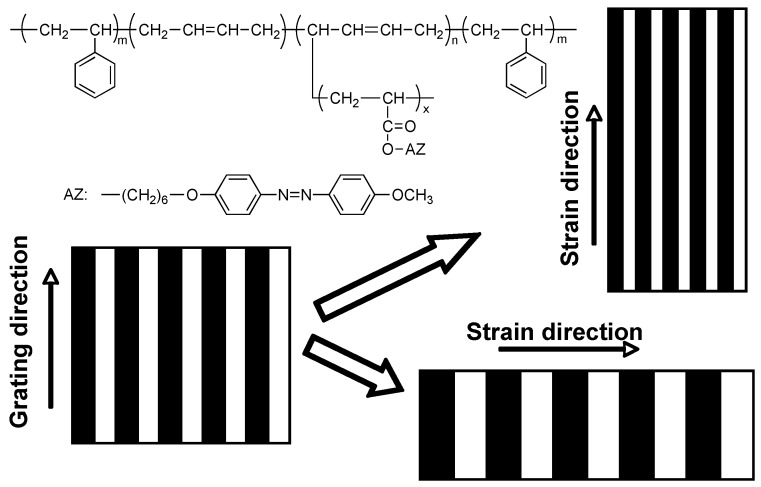
Mechanically tunable fringe spacing of gratings recorded in an ABA-type PRBC with thermoplastics.

Being a commercially-available products, ABA-type triblock copolymer poly(styrene-b-butadiene-b-styrene) (SBS) is famous for its thermoplastic properties. The hard block of PS with a content of 20–30 wt % forms the minority phase upon microphase separation, which acts as physical crosslinks for the majority phase of the soft block of rubbery polybutadiene (PB). Mechanical stretching can induce a large elastic deformation with recoverable properties. By applying this concept, Zhao *et al*. first prepared PRBCs with thermoplastics [101,102,103]. Upon stretching-induced elastic deformation of grating samples recorded in the thermoplastic PRBCs, fringe spacing (Λ) or grating periodicity was adjusted, as shown in Figure 19.

Generally, the fringe spacing could be decided by the pattern of the used photomask, when the grating is recorded with one writing beam. On the other hand, holographic gratings also can be inscribed in PRBC films by two coherent laser beams with an equal intensity, and the recorded fringe spacing can be evaluated by Λ = λ_w_/(2sinθ), where λ_w_ and θ are the wavelength and the incident angle of the writing laser beams, respectively. Once the writing beams are obtained, the fringe spacing is fixed. Tunable features of the fringe spacing were achieved using the thermoplastic PRBC (Figure 19). When the strain direction was parallel to the grating direction, the fringe spacing was decreased. On the contrary, the fringe spacing was increased when the strain direction was perpendicular to the grating direction. By the mechanical stretching, DE of the gratings was adjusted accordingly [102]. Recently, mechanically tunable fringe spacing was also obtained in gratings recorded with ABA-type triblock copolymers showing properties of conventional thermoplastic elastomers, in which rubbery poly(n-butyl acrylate) was designed as middle soft block and PRP acted as hard block [22].

#### 5.1.4. Volume Storage

**Figure 20:**
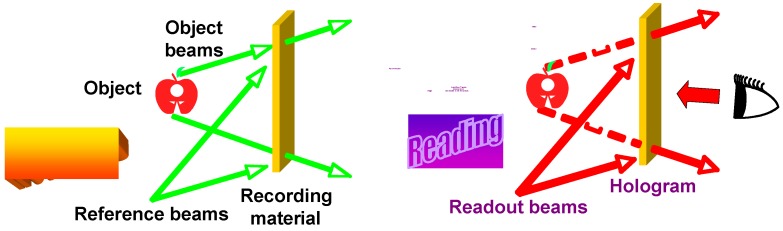
Schematic illustrations of recording and reading processes in volume storage based on PRBC materials.

In recent years, advanced recording media with fast data transfer and high density were developed. Optical holography provides unique opportunities for the next-generation storage technique (Figure 20). The storage process is based on a photoinduced refractive-index change in materials bearing chromophores. Desirable organic materials for holographic storage should exhibit high DE, fast response, high resolution, stable and reversible storage, low energy consumption during the recording and reading processes in addition to easy mass production. PRPs, photorefractive polymers, polymer-dispersed LCs, LCs or glassy oligomers were extensively investigated. But none of them could meet all the above-mentioned requirements. 

To increase storage density, Bragg-type gratings are often required. Generally, thick films (>100 μm) with low absorption and no scattering of visible light are ideal for volume holograms. But this cannot be applied directly in Bragg gratings, since it is difficult for visible light to pass through chromophore-containing thick films (eg., 100 μm) because of the large molar extinction coefficient of chromophores. One of approaches related to solve this problem is to use the cooperative effect. Ikeda *et al*. reported the formation of holographic gratings in thick films by modulation of the refractive index (*Δn*′), which was induced by the orientation of AZs and mesogens such as cyanobiphenyl or tolane moieties [104,105,106]. The random copolymers showed a maximum DE of 97% in the Bragg regime. Although 55 holograms with angular multiplicity were successfully recorded [104], the scattering could not be completely avoided.

In microphase-separated PRBCs with PRP segment constrained in the nanoscaled minority phase, elimination of the scattering and maintaining the photoinduced change in refractive index could be obtained [20,21]. Schmidt *el al*. first used this for volume storage in PS-based diblock random copolymers [107]. In the minority phase containing mesogenic segments, AZs and benzoylbiphenyl mesogenic side groups were in a statistical distribution. Thus it was possible to decrease the overall optical density, which plays an important role in the grating recording, while increasing the local refractive-index difference between illuminated and unirradiated volume elements and improving the stability of the orientation. Furthermore, they prepared thick transparent films (1.1 mm) by blending PRBCs with PS homopolymers [108], in which they performed angular multiplexing of 80 holograms at the same spatial position, showing a long-term stability at room temperature. 

### 5.2. Nanotemplates

**Figure 21:**
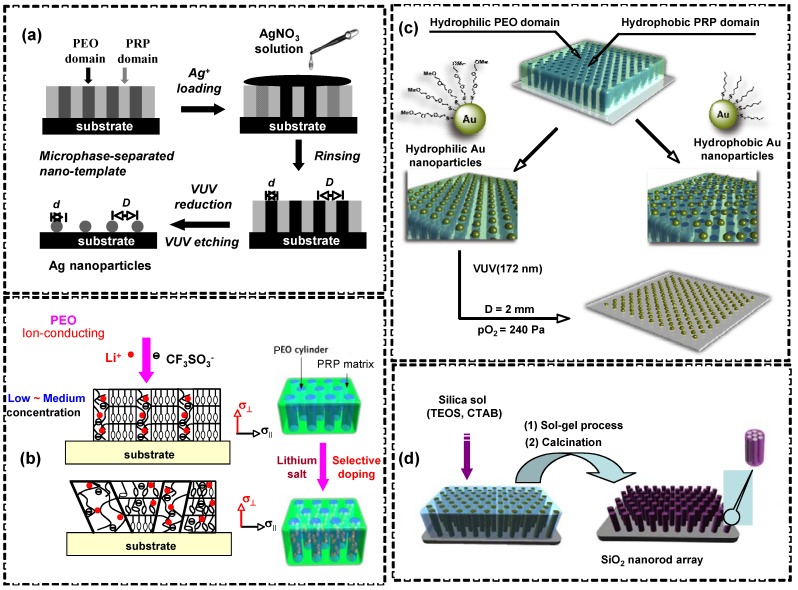
Nanotemplate applications of PRBCs. (a) Fabrication of periodic array of Ag nanoparticles. (b) Anisotropic ionic conduction in nanochannels. (c) Selective absorption of Au nanoparticles. (d) Preparation of SiO_2_ nanorod arrays by combination of a sol-gel process with PRBC lithography.

Fabrication of a well-arranged array of metal nanoparticles by using nanotemplates is one of the most important topics in nanotechnology. The size and the periodicity of the nanoparticle array can be controlled independently by choosing appropriate templates, as achieved by using well-ordered nanotemplate films of PRBCs [65]. In Figure 21a, a well-ordered array of Ag nanoparticles was successfully fabricated over a large area via selective Ag^+^-doping of the hydrophilic PEO domains in a microphase-separated PRBC film and an associated vacuum ultraviolet (VUV) treatment to get rid of the templates and simultaneously reduce the Ag^+^. Obviously, the periodicity of the high-dense Ag nanoparticles was precisely controlled by the nanotemplates of the PRBC films. It was reported that the nanotemplated fabrication of a metal nanoparticle array using the novel type of PRBC photolithography overcame the size limitation of conventional top-down lithography. Macroscopically fabricated hierarchical nanopatterns with controlled ordering indicated their potential applications ranging from photonics and plasmonics to metal wiring in molecular electronics [65,109]. The self-assembly from PRBC nanotemplates also provided a good method to modify on the nanoscaled shape of various kinds of functional materials, such as electric conducting RuO_2_, magnetic Fe, or organic conducting polymers [109]. 

In addition, anisotropic PEO nanocylinder arrays were used as ion conductive channels since PEO has been widely used as solid electrolyte. In Figure 21b, a supramolecularly complexed structure and anisotropic ion transportation were achieved based on PRBC nanotemplates by incorporating LiCF_3_SO_3_ into the PEO nanocylinders [66]. Highly ordered ion-conducting nanocylinder channels with perpendicular orientation were formed by coordination between the lithium cations and the ether oxygens of the PEO blocks. At low and medium salt concentrations, selective complexation of Li^+^ with the PEO phase led to the formation of an ordered array of ion-conducting PEO nanocylinders. At high salt concentration, the lithium salt dissolved in both PEO and the AZ domains decreases LC ordering and disturbed microphase separation. As a result, tilted and distorted nanocylinders were formed with poor regularity, and the anisotropic value of ion conductivity was reduced [66].

On the surface of PEO-based PRBC films, each hydrophilic PEO domain appears as a circular hollow surrounded by the hydrophobic PRP matrix. These amphiphilic properties enabled the selective absorption of Au nanoparticles with hydrophilic or hydrophobic modifications with functional ligands. The surface property of Au nanoparticles was a critical factor in this nanofabrication [110]. In Figure 21c, site-specific recognition of Au nanoparticles was obtained in PEO nanocylinders or the continuous domains formed with the PRP block. Then the ordering of the gold nanoparticles from the nanotemplate was transferred to the substrate by a VUV approach.

In sol-gel processed incorporated with PRBC lithography, a hexagonal array of ordered SiO_2_ nanorods with mesochannels aligned along the longitudinal axes was obtained in Figure 21d [111]. The mesochannels inside the SiO_2_ nanorods were aligned perpendicularly to the substrate and had a diameter of about 2 nm. The height of hierarchically ordered mesoporous silica of several-hundred nanometers was achieved, indicating their potential applications as innovative materials for waveguides, lasers, and biomacromolecular separation systems.

The amphiphilic PRBC with well-ordered microphase separation shows excellent reproducibility and mass production through molecular or supramolecular self-assembly. Both parallel and perpendicular patterns of nanostructures with high regularity can be precisely manipulated. This guarantees the nanotemplate-based fabrication processes, and results in formation of diverse self-assembled nanostructures, leading to industrial applications in plastics engineering.

### 5.3. Photocontrolled Deformation

As above-mentioned, the photoresponsive properties enabled the microphase-separated structures in PRBC films to be 3D photocontrollable. Similar photoinduced phenomena were also found in well-defined PRBCs in different states, such as Langmuir-Blodgett (LB) films [112], micelle [26,27,113,114,115,116,117,118,119,120,121], colloidal spheres and elastomers [50,53,122,123,124]. 

Seki *et al*. prepared LB films using an ABA-type PRBC with AZs as photoactive moieties, which was then transferred onto a freshly-cleaved mica surface by vertical dipping method [112]. The thickness of the monolayer film was in a molecular size, but the area could be expanded to a macroscopic scale. It was found that active and quasi-reversible photocontrol of two-dimensional (2D) microphase separation was induced by photoisomerization of AZs. 

**Figure 22:**
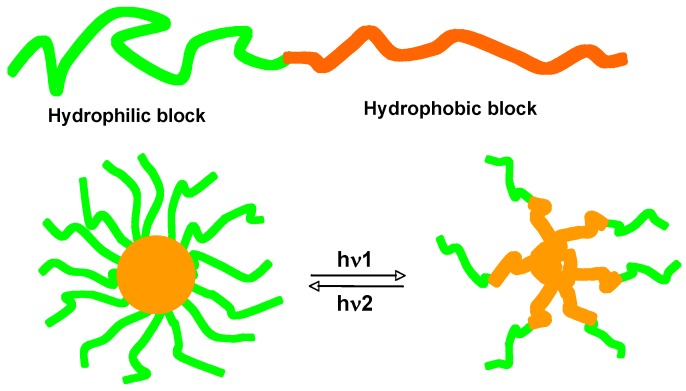
Schematic illustration of reversible dissociation and formation of PRBC micelles upon photoirradiation.

In aqueous solution, amphiphilic BCs often forms micelles when the concentration is higher than their critical micelle concentrations (CMCs), and a variety of morphologies can be self-organized by micellar aggregation [26]. Photoresponsive micelles can be fabricated with amphiphilic PRBCs, as shown in Figure 22. Zhao *et al*. reported reversible dissociation and formation of micelles induced by photoisomerization of AZs, which was attributed to the photoinduced change in dipole moments of chromophores [27,113]. Similarly, reversible disruption and regeneration of PRBC micelles was achieved by reversible photoreaction of spiropyran chromophores upon irradiation with UV and visible light [114]. With irreversible photocleavage of dyes, light-breakable PRBC micelles were also investigated [115,116]. Functionalized PRBC micelles with high stability were obtained by using the photodimerization of cinnamates or coumarins after micelle formation [117,118,119,120,121].

Recently, interesting photoresponsive behaviors of PRBCs was found in solid state. Using amphiphilic PRBCs, uniform spherical aggregates were obtained by gradually adding water into its THF solution, which could be significantly elongated in the polarization direction of the actinic light [50,52]. Combing with SRG formation on the PRBCs, Wang *et al*. pointed out that the flexible spacer in the PRP block could play an important role in transferring thelight-driving force from chromophores to polymer backbones [122]. On the other hand, Li *et al*. reported light-responsive nematic LC elastomer fibres with AZ-containing PRBCs by incorporating 5CB as a plasticizer [123,124]. Coinciding with LC elastomers reported by Ikeda *et al*. [11], the PRBC fibres bent towards the direction of the incident UV light, indicating their potential applications as artificial muscles. 

### 5.4. Microporous Structures

**Figure 23:**
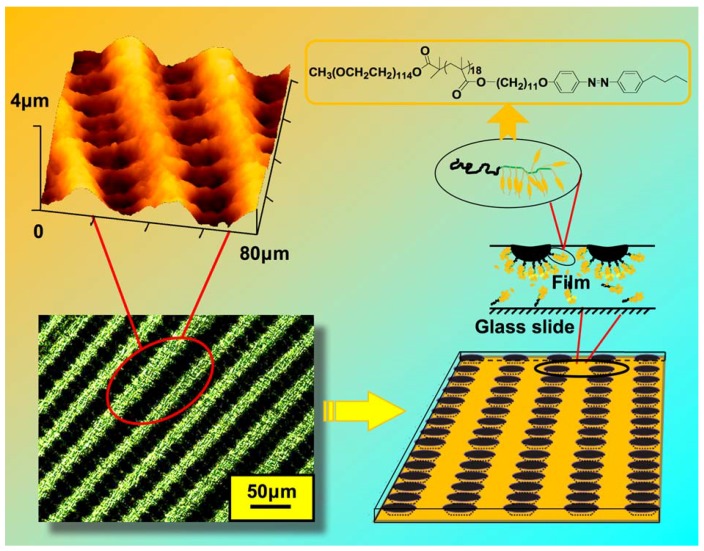
Fabrication of regularly patterned micropores with an amphiphilic PRBC by spin coating under a dry environment.

With an amphiphilic PRBC consisting of a flexible PEO segment as a hydrophilic part and poly(methacrylate) containing an AZ moiety in side chain as a hydrophobic one, well-arranged ellipsoidal micropores embedded in an LC matrix were fabricated by spin coating under a dry environment (Figure 23) [125]. The formation process of the microporous films consisted of the following steps: Firstly, the PRBC was dissolved in tetrahydrofuran (THF), a good solvent for both segments. To avoid humid condition, a small amount of water was added to the THF solution. Then the water-containing THF solutions were spin coated on clean glass slides. Upon spin coating, the evaporation of THF cooled the PRBC surfaces down and led to the formation of water droplets, which were stabilized by the amphiphilic PRBC and packed periodically. At the final step, the regularly patterned microporous films were obtained after complete evaporation of water and THF.

With the help of small amount water, the obtained pore size was controlled in a range of several-ten microns [125]. Then the influence of water content and rotational speed was studied in detail. It was found that regularly patterned microporous films could be prepared with certain water content, and the porous size could be easily tailored with changing the rotational speed. The obtained microporous structures showed good thermal stability below the LC-to-isotropic phase-transition temperature of the PRBC. Similarly, even the photoinduced LC-to-isotropic phase transition was induced upon UV irradiation at room temperature, the fabricated micropores showed no change in size upon photoirradiation. Although the mesogens in the PRBC films were randomly distributed, the LC property of self-organization might play an important role in the formation of linearly patterned ellipsoidal micropores embedded in a birefringent LC matrix with photoresponsive functions. 

## 6. Conclusions

The microphase-separated nanostructures of well-defined PRBCs have fascinated one to understand the relationship between their ordered structures with photocontrollable properties of PRP blocks. One of the most exquisite advantages of introducing PRPs into well-defined PRBCs is precise photo-manipulation of the supramolecularly self-organized nanostructures in PRBCs. With the development of information technology, new waves are surging, driving such nanostructures leading to industrial applications as the future engineering plastics for optoelectronics and nanotechnology. Being expected as one of the powerful counterparts of top-down-type nanofabrication, the central focus should be placed on high reproducibility and mass production as well as precise manipulation of these ordered nanostructures. Although the research on PRBCs is still in a primary stage, many groups are involving in this novel field of PRP materials, which will improve our understanding of functional PRBCs and push ahead to find their diverse applications in optoelectronics, information storage, nanotechnology, as well as biotechnology.
